# Make up a missed lesson‐New policy to ensure the interchangeability of generic drugs in China

**DOI:** 10.1002/prp2.318

**Published:** 2017-05-03

**Authors:** Baobin Huang, Sarah L. Barber, Mingzhe Xu, Shuanghong Cheng

**Affiliations:** ^1^ Division of General ManagementNational Institutes for Food and Drug Control and WHO Collaborating Centre for Drug Quality AssuranceNo.2, TiantanxiliDongchengBeijingChina; ^2^ Health System SpecialistWHO Representative Office in South Africa351 Francis Baard (Schoeman) StreetPretoriaSouth Africa; ^3^ Present address: World Health Organization Centre for Health Development 1‐5‐1 Wakinohama‐KaigandoriChuo‐kuKobe651‐0073Japan

**Keywords:** Bioequivalence studies, clinical trial, comparators, drug regulation, efficacy, essential medicines, generic drugs, generic drugs industry, interchangeability, quality, re‐evaluation

## Abstract

Generic drugs should be interchangeable with originators in terms of quality and efficacy. With relative lower prices, generic drugs are playing an important role in controlling health expenditures and ensuring access. However, the widespread understanding of “cheap price equals low quality” has a negative impact on the acceptance of generic drugs. In China, medical doctors doubt the efficacy and quality of generic drugs manufactured domestically. To address these concerns, the Chinese State Council released a policy in 2016 to ensure the interchangeability by re‐evaluating the quality and efficacy of generic drugs. It intends to make up a missed lesson in the regulation to be in line with internationally accepted practices. Generic drugs firms, depends on the availability of appropriate comparators, should conduct either comparative bioequivalence studies or full scale clinical trials. The re‐evaluation will be implemented in a stepwise approach with the essential medicines covered in the first step. The policy could achieve several benefits by increasing confidence on the Chinese produced generic drugs, upgrading regulatory standards, streamlining the Chinese generic drug industry and creating a healthy competition market. Nevertheless, enormous challenges remain in enlarging the capacity to review applications, selecting appropriate comparators, ensuring the capacity of domestic clinical research sites, and achieving the acceptance of re‐evaluated generic drugs.

## Introduction

On Feb 6, 2016, the General Office of the Chinese State Council (i.e., the Cabinet) released a new policy on the re‐evaluation of quality and efficacy of registered generic medicines (General Office of Chinese State Council 2016). It marked the upgrading of regulatory standards on generic drugs by referring to international accepted practices (China Pharmaceutical News 2016). The re‐evaluation is a watershed moment for domestic generic drugs firms – faced with the choice of re‐ensuring the interchangeability of their generic products in line with the currently upgraded regulatory standards or withdrawing from the market.

From the perspective of drug regulation, a generic drug should be interchangeable with the originator drug in the aspects of quality, clinical efficacy, and safety (European Medicines Agency 2012; U.S. Food and Drug Administration 2015). This initiative intends to make up a missed lesson in the development course of generic drugs regulation in China. The reform of the drug review system in China was launched in 2000, from which assessing clinical interchangeability with recognized comparators was not a compulsory requirement (State Council Information Office 2016). The similar work for re‐ensuring the interchangeability of generic drugs with originator drugs has been or is being done by the regulatory bodies of some developed nations, such as Japan and U.S. Generic drugs are usually sold at lower prices compared with the originator drugs, and play an important role in controlling health expenditures and ensuring access (Cameron et al. 2012). Taking the consumption of two widely used medicines–anti‐hypertensives and anti‐diabetics–in public hospitals in China between 2012 and 2014, a total of US$ 4.2 billion could be saved from switching to generic formulations (Sun et al. 2016). With quality assured generic drugs available on the domestic market, it is anticipated that a great potential savings would be achieved. However; the widespread perception of “cheap price equals low quality” has a negative influence on the confidence of both health professionals and patients, thus impeding the implementation of generic substitution in China in the future. Generic drugs are usually registered based on a single in vivo comparative bioavailability study for the demonstration of bioequivalence to the originator drug. Without clinical interchangeability demonstration based on comparable clinical efficacy and safety studies, the bioavailability of generic drugs could be sensitive to a difference in quality properties, affecting the interchangeability of a generic and originator drug. In the Asian Pacific region, lack of clear bioequivalence assessment system contributes to the mistrust in the quality of generic drugs (Nguyen et al. 2013). Specifically in China, medical doctors doubt the efficacy and the quality of generic drugs manufactured domestically. Overall, strengthening the regulation of generic drugs was therefore essential to address concerns related to the quality of generic drugs.

In the new policy in 2016 for re‐evaluation of generic drugs, the pharmaceutical companies basically are requested to conduct comparative studies to demonstrate bioequivalence between their generic drugs and the originator drugs, in order to maintain or renew the license. If available, the originator drugs should be used as comparators for the first choice, otherwise the “well‐established” generic drugs as comparators can also be accepted. The *in vivo* comparative bioavailability studies or the *in vitro* comparative dissolution tests should be conducted depending on whether bioequivalence studies can be waived. If comparators are not available, a full scale clinical efficacy and safety trial should be conducted. Once the renewing application with the requested studies is submitted, the development and production sites will be inspected and three consecutive batches will be tested to control the quality of the drug products by the corresponding institutions of drug regulatory authority in China, e.g. the National Institutes for Food and Drug Control.

All generic drugs registered currently by the Chinese Food and Drug Administration for which bioequivalence with a recognized comparator drug has not been reviewed should participate in the re‐evaluation. The re‐evaluation will be implemented in a stepwise approach. In the first phase, the re‐evaluation for the chemical drugs with an oral solid dosage form registered before October 1st, 2007 and listed in the National Essential Medicine List (2012 edition) is planned to be complete by the end of 2018. For the generic drugs without a comparator, a three‐year extension can be granted for conducting the full scale clinical efficacy and safety trials. It is estimated that approximately 1900 pharmaceutical companies and 18,000 generic drugs should participate the re‐evaluation procedure to ensure the quality of generic drugs in China over the next three to five years. In addition, the re‐evaluation steps forward in the alignment with the stringent regulatory bodies. If a generic drug has also been registered in Europe, U.S., or Japan, the applicant can apply to renew registration for the generic drug in China without conducting additional comparative studies. Once the renewal registration is approved, the generic drugs manufactured in the same production line would also be considered bioequivalence with the originator drug and can be accepted.

Via the re‐evaluation of generic drugs in China, several benefits can be expected. First, we expect an increased confidence in the quality of domestic generic drugs by the public. Second, Chinese regulation on generic drugs will move toward international standards. Third, the implementation of the re‐evaluation policy could build confidence of the international community on Chinese made generic drugs and make them more competitive in the international markets. Fourth, the Chinese generic companies will be streamlined and standardized by forcing to remove low quality and excessive production lines. Fifth, the enforcement of re‐evaluation could create a “quality oriented” healthy market environment and reduce the pressure for generic producers to use unhealthy competition measures such as financial inducement. Nevertheless, enormous challenges remain in implementing the re‐evaluation and promoting utilization of generic drugs. For carrying out the re‐evaluation, these challenges include enlarging the capacity of the Chinese drug regulatory body to review applications; ensuring the availability and selection of appropriate comparator drugs; and ensuring the capacity of domestic clinical research sites for conducting clinical trials. With regards to the utilization of quality assured generic drugs in China healthcare system, there is need to address the misunderstanding of the public and health professionals on generic drugs. This can be achieved through education to the public/consumers and health professionals, such as incorporating generic drugs related topics into the curricula or training programs of pharmacy colleges (Alrasheedy et al. 2014; AL‐Tamimi et al. 2016). Despite these obstacles, the policy represents a critical first step towards improving the quality of generic drugs produced in China and lays the solid foundation for acceptance and utilization of generic drugs in next steps.

## Disclosure

None declared.
